# A Case Series of Four Patients with Artery of Percheron Occlusion over a Three-Month Period

**DOI:** 10.3390/neurolint15040085

**Published:** 2023-11-02

**Authors:** Matej Perovnik, Janja Pretnar Oblak, Senta Frol

**Affiliations:** 1 Department of Vascular Neurology, University Medical Center Ljubljana, 1000 Ljubljana, Slovenia; matej.perovnik@kclj.si (M.P.); janja.pretnar@kclj.si (J.P.O.); 2 Faculty of Medicine, University of Ljubljana, 1000 Ljubljana, Slovenia

**Keywords:** artery of Percheron, stroke, thalamus, case series, incidence

## Abstract

Here, we present a case series of four patients diagnosed with acute ischaemic stroke due to occlusion of the artery of Percheron (AOP), a rare stroke variant, observed in a single emergency centre within a three-month period. AOP occlusion is characterized by bilateral thalamic infarction with or without involvement of the mesencephalon. The presenting symptoms are diverse and not specific, but commonly include disturbance of consciousness, memory impairment, and vertical gaze palsy. In addition, due to the location of the infarction, imaging recognition is challenging and AOP occlusion often remains undiagnosed. This paper emphasizes the necessity of early recognition and appropriate management of AOP occlusion to significantly impact patient outcomes. Moreover, we argue that the condition might be more common than previously thought and that misdiagnosis or delay in diagnosis may lead to inappropriate treatment and potential failure to apply thrombolysis within the required timeframe.

## 1. Introduction

Even with the advancement of diagnostic and treatment options, acute ischaemic stroke (AIS) treatment is still based on prompt and correct clinical diagnosis. Compared to the anterior circulation, clinical presentation of AIS in the posterior circulation is much more diverse [1]. In this regard, the occlusion of the artery of Percheron (AOP), the anatomical variant of the posterior circulation, is one of the most challenging. Without prompt treatment, it results in bilateral thalamic infarction with or without the involvement of the mesencephalon [2,3] and, consequently, high morbidity and mortality, especially in the former [2]. Although AOP occlusion is related to diverse prognostic outcomes, around half of the patients have unfavourable functional outcome with a modified Rankin scale (mRs) score of 3 and more [2].

The data about AOP prevalence are not well known. According to the post mortem examinations, AOP is present in approximately 10% of the adult population [3]. However, AOP occlusion is a rare cause of AIS, and a recently published retrospective single centre study reported incidence of 0.53% [4]. Similarly, other large retrospective stroke series reported the incidence of AOP infarction to be between 0.27 and 0.80% [2,4,5,6,7], Table 1.

## 2. Case Presentation

Here, we describe four cases of AOP occlusion that were diagnosed in a single Neurology Emergency Department (NED) of a University Medical Centre within three months between March and June 2023, Table 2.

### 2.1. Case 1

**Figure 1:**
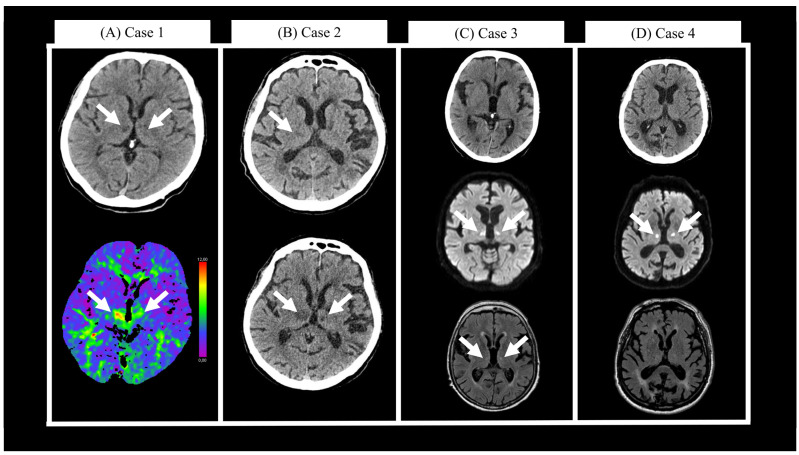
(**A**) Case 1: head computer tomography (CT) (upper) and CT perfusion (lower) scans two days after initial presentation. (**B**) Case 2: head CT scans at initial presentation (upper) and one day later (lower). (**C**) Case 3: head CT scan at initial presentation (upper), magnetic resonance imaging (MRI) diffusion weighted imaging (middle), and fluid attenuated inversion recovery (lower) at initial presentation. (**D**) Case 4: head CT (upper), MRI diffusion weighted imaging (middle), and fluid attenuated inversion recovery (lower) at initial presentation. White arrows show changes in the thalami.

A 70-year-old male with a history of alcoholism and homelessness presented to the Internal Medicine Emergency Department (IMED) due to instability while biking. Electrocardiogram (ECG) showed a known atrial fibrillation (AF) with tachycardia (146/min), and he had elevated blood ethanol levels (61.1 mmol/L, 2.2‰). He received saline infusion and was dismissed. The next day he was found in a local store, hypoglycaemic and somnolent with Glasgow Coma Scale (GCS) 7. At the IMED, his ECG once again showed AF with tachycardia (114/min). He had elevated ethanol blood levels (60.6 mmol/L, 2.2‰) and mild hyponatremia (130 mmol/L). As the somnolence persisted until the next morning, a head computer tomography (CT) scan was performed, which showed chronic vascular leukopathy and two hypodensities in the right thalamus and right corona radiata. He was transferred to the NED, where he was somnolent and severely dysarthric with GCS 9. A repeated head CT showed hypodensities in bilateral medial thalami that extended to the anterior mesencephalon (Figure 1A, upper), while CT perfusion (CTp) showed decreased hypoperfusion in the same areas (Figure 1A, lower). CT angiography (CTA) was unremarkable. The next day, at the ward, he was less somnolent, still dysarthric without language deficits, he had vertical gaze palsy, wider right pupil, right ptosis and left sided hemiparesis. His hospitalization was prolonged due to homelessness. The patient was dismissed after 64 days. He was moderately dysarthric with better comprehensiveness, he had vertical gaze palsy and ataxia in the left limbs with positive Romberg test. His National Institutes of Health Stroke Scale (NIHSS) score was four and mRS was one. Anticoagulation treatment was not initiated due to history of repeated falls, alcoholism, and lack of adherence.

### 2.2. Case 2

A 74-year-old male with previous history of cognitive decline presented to the ED due to one week of general weakness. He was febrile (38.3 °C), tachycardic (103/min), and had mild infiltrative lesions on chest x-ray. Antibiotic treatment was started due to suspected pneumonia. Two days later, he was brought to IMED due to impaired consciousness, GCS 7. A head CT showed only older lacunar ischaemic lesions in the right thalamus, right putamen, and left internal capsule (Figure 1B, upper). Chest x-ray did not show worsening of pneumonia. He was referred to the NED where he was reacting only to pain stimuli. A repeated head CT showed enlargement of the hypodensity in the right anterior thalamus. Cerebrospinal fluid (CSF) analysis was normal. He was admitted to the ward, where disturbance of consciousness persisted, and a third head CT showed bilateral infarction of medial thalami (Figure 1B, lower). CTA was unremarkable. His pneumonia worsened clinically and radiologically, and in addition, he suffered a subacute myocardial infarction. Initial treatment with amoxicillin–clavulanate was switched to piperacillin/tazobactam. At the ward, he was somnolent with short spells of spontaneous eye opening, but no meaningful communication was possible. He died 55 days after the initial presentation to the ED.

### 2.3. Case 3

A 73-year-old female was brought to the NED due to acute disturbance of consciousness, GCS 7. She was only reacting to painful stimuli without lateralization. Her ECG revealed an AF with normocardia. Blood work was unremarkable, but chest x-ray showed bilateral pleural effusion and atelectasis without inflammation. Head CT did not show any acute or subacute ischaemic areas (Figure 1C, upper); likewise, CTp and CTA did not reveal any significant pathological findings. Due to suspected seizure, a combination of levetiracetam and lacosamide was administered, but her consciousness gradually worsened, and her right pupil slightly widened. A repeated head CT did not show any changes. Magnetic resonance imaging (MRI) was performed, which revealed an acute ischaemic lesion (Figure 1C, middle) bilaterally in medial thalami, anteromedial mesencephalon and in periaqueductal grey matter. The fluid attenuated inversion recovery (FLAIR) sequence showed two small hyperintensities in bilateral medial thalami (Figure 1C, lower). Her condition spontaneously improved, and one day later, she was awake and alert. Neurological examination revealed vertical gaze palsy, diplopia, rest tremor, orofacial dyskinesia, and mild ataxia in all four limbs. Six days after the initial presentation, a repeated head CT showed only a demarked lesion in the left medial thalamus. There was no haemorrhagic transformation, and apixaban treatment was initiated due to AF. At discharge, 12 days later, she scored four both on NIHSS and mRS. At the follow-up examination 3 months after discharge, her mRS score was three.

### 2.4. Case 4

An 81-year-old male presented to the NED due to disturbance of consciousness. His symptoms began the previous evening with transient diplopia and gait instability. Next morning at NED (9 h after first presentation), he was somnolent, first reacting only to painful stimuli but later also to verbal command. ECG was normal. Head CT showed only older ischaemic lesions in occipital and parietal cortex and right lateral thalamus (Figure 1D, upper). CTp showed decreased perfusion bilaterally in the ventral thalami and upper mesencephalon. CTA was unremarkable. MRI showed diffusion restriction in the same areas (Figure 1D, middle), and since no changes were seen on the FLAIR sequence (Figure 1D, lower), an intravenous thrombolysis was administered. He was admitted to the ward where he gradually became more alert but dysarthric. A vertical gaze palsy (more pronounced upward) and limited adduction in the left eye was observed. At the ward, he was also treated for bilateral conjunctivitis, lower urinary tract infection, and mild parkinsonism. At discharge, 24 days later, he was still disoriented in time, had memory impairment, and gaze palsy; he scored five both on NIHSS and mRS. At the follow-up examination 3 months after discharge, his mRS score was four.

**Table 2:** Patients’ demographics and clinical characteristics.

Patient	Sex	Age	Time from Symptom Onset to Arrival [h]	Time from Arrival to Diagnosis [h]	Presenting Symptoms	Infarction Topography	Acute Treatment	mRS after 3 Months
1	M	70	1	25	Somnolence; vertical gaze palsy; dysarthria; gait instability	Paramedian bilateral thalami; anterior mesencephalon	LMWH, Aspirin	1
2	M	74	1	21	Impaired consciousness	Bilateral medial thalami	LMWH, Aspirin	6
3	F	73	1.5	7	Somnolence; right pupil widening; vertical gaze palsy; diplopia; orofacial dyskinesia; mild ataxia in all four limbs	Bilateral medial thalami; anteromedial mesencephalon	Apixaban	4
4	M	81	13.5 (TIA), wake-up stroke	0.5	Diplopia; gait instability; somnolence; vertical gaze palsy	Bilateral medial and ventral thalami; superior mesencephalon	IVT, aspirin, clopidogrel	4

M—male; F—female; LMWH—low-molecular-weight heparin; mRS—modified Rankin Scale.

In addition to the four presented cases, we conducted a search of head CT and MRI radiological reports from scans performed within the last 5 years (between 22 July 2018 and 22 July 2023) searching for the mention of AOP occlusion. We identified just six more patients with AOP occlusion that were treated at University Medical Centre Ljubljana (UMCL) in the preceding five years. Single cases were recognized in years 2018, 2020, and 2021, and three in 2022. In 2018, we treated 1 patient with AOP occlusion out of 1121 patients with AIS (1/1121 = 0.09%), as was the case in 2020 (1/1003 = 0.10%) and in 2021 (1/981 = 0.10%). In 2022, there were 3 cases with AOP occlusion out of 1056 treated AIS patients (3/993 = 0.30%). The estimated prevalence for the first half of year 2023 was 1.53%.

## 3. Discussion

In this paper, we presented four patients with AOP occlusion that were treated at our UMCL over a short course of three months. The patients had variable outcomes, highlighting the need for increased awareness and understanding of this condition. Misdiagnosis or delay in diagnosis can lead to missing the time window for the thrombolysis and inappropriate treatment. Through these cases, we aimed to emphasize that early recognition and appropriate management of AOP occlusion can significantly impact patient outcomes.

Recognition of acute AOP occlusion is a clinical challenge. Non-specific symptoms can be explained by the different roles of the thalamus. The thalamus is a bilateral structure located between the brainstem and the telencephalon which plays an important part not just in relaying [8] but also in shaping and modulating brain functional networks associated with arousal, cognition, and conscious awareness [9]. Therefore, the infarction in this area due to AOP occlusion can lead to a wide range of neurological and cognitive deficits. The classical triad of symptoms related to AOP occlusion include altered mental status, memory impairment, and vertical gaze palsy [10].

**Figure 2:**
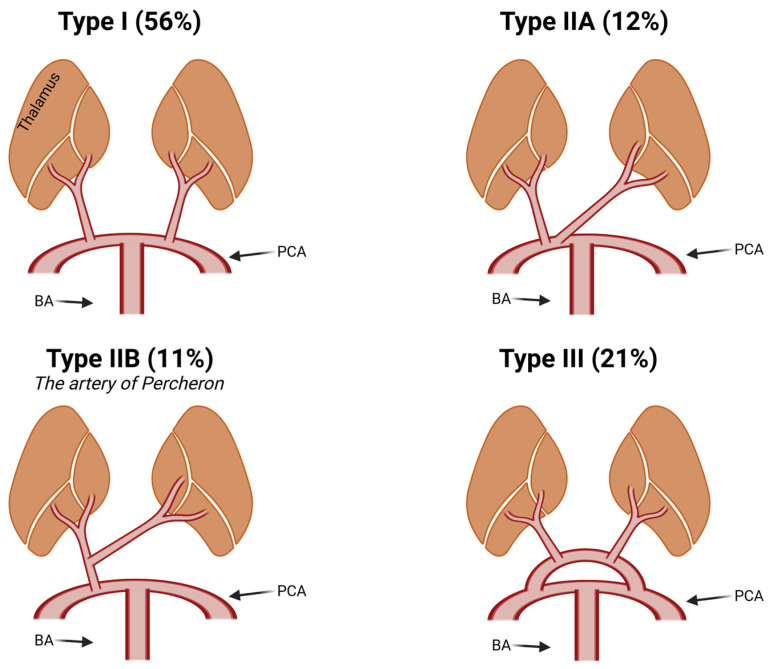
Schematic depiction of the thalamus and its arterial supply according to the Percheron classification [11] and the estimated population prevalence of each variant [3]. PCA—posterior cerebral artery, BA—basilar artery. Created with BioRender.com (© 2023 BioRender).

Variability of the clinical presentation is also a consequence of variable arterial supply of the paramedian thalamic area. In 1973, Gerard Percheron described four variants (types I, IIa, IIb, and III), Figure 2 [11]. AOP corresponds to type IIb which occurs in about 11% of the population [3]. In the case of AOP, a single artery arises from one posterior cerebral artery (PCA) and then bifurcates to supply the bilateral thalami causing a bilateral paramedian thalamic infarction [11,12]. Even within this subtype, there are four distinct ischaemic patterns: (i) with involvement of mesencephalon, (ii) without involvement of mesencephalon, (iii) with involvement of anterior thalamus and mesencephalon, and (iv) with involvement of anterior thalamus without mesencephalon [13]. The most common are the first two patterns which corresponded to 81% of the cases in the study by Lazzaro et al. [13]. Three of our patients (cases 1, 3, and 4) belong to the (i) group and one (case 2) to the (ii) group. Involvement of the mesencephalon often leads to additional symptoms, such as cerebellar ataxia, oculomotor disturbances, and hemiplegia [13].

In addition to variable clinical presentation, acute AOP occlusion also presents an imaging challenge. Similar to our case series, Flowers et al. [14] reported three cases of AOP infarction seen over three months in 2021. As in the case series presented in this paper, all three CT scans were initially negative, and MRI was necessary to make a final diagnosis. Although in most cases MRI enables acute diagnosis by revealing a distinct pattern of V-shaped hyperintensity on FLAIR and/or DWI [13], it may not always be available in the acute emergency setting. Alternatively, a CTp, as in our Cases 1 and 4, can be utilized. CTp is a widely available method used to visualize the core of the infarction and salvageable penumbra. However, due to technical limitations, CTp has a limited brain coverage and an exact clinical question is needed to correctly position the window to capture the area of thalamus and mesencephalon [15]. Furthermore, the diagnostic accuracy of CTp is further reduced in posterior circulation stroke [16]. Even though several automated semi-quantification tools, such as RAPID (RapidAI^®^, San Mateo, CA, USA), VIZ CTP (Viz.ai, San Francisco, CA, USA), e-Mismatch (Brainomix, Oxford, United Kingdom) or syngo.via (Siemens Healthineers, Erlangen, Germany), have the potential to improve diagnostic accuracy, they are still dependent on the position of the window. Whole-brain CTp can overcome that issue, but so far, it is also not widely available [17].

Taken together, our cases as well as data from the literature suggest that a higher vigilance among neurologists, internal medicine physicians, and radiologists is necessary to recognize and appropriately treat these patients. Delayed diagnosis leads to missing the time window for thrombolysis and, thus, to possible worse outcomes. In our case series, only one patient (case 4) received thrombolytic treatment; however, his outcome was nonetheless poor. Of note, this patient received thrombolytic treatment several hours after initial symptom onset on the basis of DWI-FLAIR mismatch [18]. The initial symptoms were transient, and we can speculate that the complete AOP occlusion causing the disturbance of consciousness appeared during the night. Thrombolysis in AOP occlusion has been shown to be beneficial before. In a series of 23 patients presented by Zhang et al. [19], 6 patients received thrombolysis and all of them had a good outcome at 90 days (mRS ≤ 2). However, none had additional involvement of the midbrain, as seen in our case 4. It has been shown before that patients with additional rostral midbrain infarctions have worse outcomes as compared to patients without [2].

## 4. Conclusions

Here, we report four patients with AOP infarction seen within three months at a single UMC. The short time in which the cases presented highlights the possibility that AOP infarctions are more common than previously thought and the condition might be overlooked as the patients with disturbance of consciousness are not necessarily first referred to the Neurology Clinics. Additionally, as observed in all four cases, acute CT imaging can be unremarkable and targeted CTp or MRI should be used. Consequently, AOP infarction is diagnosed only by the follow-up CT or MRI when the changes are already irreversible or not at all.

## Figures and Tables

**Table 1:** Overview of large stroke series that studied the prevalence of artery of Percheron (AOP) occlusion.

Study	AOP Cases	Total Sample	
Carrera et al. [5]	10	3712	0.27%
Caballero [6]	10	1253	0.80%
Arauz et al. [2]	15	3750	0.40%
Xu et al. [7]	18	6539	0.28%
Ciacciarelli et al. [4]	15	2830	0.53%

## Data Availability

All the data generated or analysed during the study are included in this article. Further inquiries can be directed to the corresponding author.

## References

[1] Nouh A., Remke J., Ruland S. (2014). Ischemic Posterior Circulation Stroke: A Review of Anatomy, Clinical Presentations, Diagnosis, and Current Management. Front. Neurol..

[2] Arauz A., Patiño-Rodríguez H.M., Vargas-González J.C., Arguelles-Morales N., Silos H., Ruiz-Franco A., Ochoa M.A. (2014). Clinical Spectrum of Artery of Percheron Infarct: Clinical–Radiological Correlations. J. Stroke Cerebrovasc. Dis..

[3] Kocaeli H., Yılmazlar S., Kuytu T., Korfalı E. (2013). The artery of Percheron revisited: A cadaveric anatomical study. Acta Neurochir..

[4] Ciacciarelli A., Francalanza I., Giammello F., Galletta K., Toscano A., Musolino R.F., Granata F., La Spina P. (2023). Prevalence, clinical features, and radiological pattern of artery of Percheron infarction: A challenging diagnosis. Neurol. Sci..

[5] Carrera E., Michel P., Bogousslavsky J. (2004). Anteromedian, Central, and Posterolateral Infarcts of the Thalamus. Stroke.

[6] Jiménez Caballero P.E. (2010). Bilateral Paramedian Thalamic Artery Infarcts: Report of 10 Cases. J. Stroke Cerebrovasc. Dis..

[7] Xu Z., Sun L., Duan Y., Zhang J., Zhang M., Cai X. (2017). Assessment of Percheron infarction in images and clinical findings. J. Neurol. Sci..

[8] Sherman S.M. (2007). The thalamus is more than just a relay. Curr. Opin. Neurobiol..

[9] Shine J.M., Lewis L.D., Garrett D.D., Hwang K. (2023). The impact of the human thalamus on brain-wide information processing. Nat. Rev. Neurosci..

[10] Schmahmann J.D. (2003). Vascular Syndromes of the Thalamus. Stroke.

[11] Percheron G. (1973). The Anatomy of the Arterial Supply of the Human Thalamus and Its Use for the Interpretation of the Thalamic Vascular Pathology. Z. Neurol.

[12] Yang F., Hung J., Lin S. (2021). Percheron Artery-Plus Syndrome: A Syndrome Beyond Stroke Chameleon. J. Nippon Med. Sch..

[13] Lazzaro N.A., Wright B., Castillo M., Fischbein N.J., Glastonbury C.M., Hildenbrand P.G., Wiggins R.H., Quigley E.P., Osborn A.G. (2010). Artery of Percheron Infarction: Imaging Patterns and Clinical Spectrum. Am. J. Neuroradiol..

[14] Flowers J., Gandhi S., Guduguntla L., Yang A., Moudgil S. (2022). Artery of Percheron Strokes: Three Cases in Three Months. Cureus.

[15] Lamot U., Ribaric I., Popovic K.S. (2015). Artery of Percheron infarction: Review of literature with a case report. Radiol. Oncol..

[16] Katyal A., Calic Z., Killingsworth M., Bhaskar S.M.M. (2021). Diagnostic and prognostic utility of computed tomography perfusion imaging in posterior circulation acute ischemic stroke: A systematic review and meta-analysis. Eur. J. Neurol..

[17] Lin L., Bivard A., Krishnamurthy V., Levi C.R., Parsons M.W. (2016). Whole-Brain CT Perfusion to Quantify Acute Ischemic Penumbra and Core. Radiology.

[18] Thomalla G., Cheng B., Ebinger M., Hao Q., Tourdias T., Wu O., Kim J.S., Breuer L., Singer O.C., Warach S. (2011). DWI-FLAIR mismatch for the identification of patients with acute ischaemic stroke within 4·5 h of symptom onset (PRE-FLAIR): A multicentre observational study. Lancet Neurol..

[19] Zhang B., Wang X., Gang C., Wang J. (2022). Acute percheron infarction: A precision learning. BMC Neurol..

